# Qualitative evaluation of a community intervention in educational centres: Opinion of the professionals involved

**DOI:** 10.23938/ASSN.1150

**Published:** 2026-04-22

**Authors:** María Clara Vidal-Thomàs, Joana Ripoll Amengual, María Belén Cacereño Jiménez, Pau Sempere Andrés, Elisa Urbina Sampedro, Sebastiá March Llull

**Affiliations:** 1 Technical Office of Primary Care Management of Mallorca Balearic Islands Health Service Palma Mallorca Spain; 2 Health Research Institute of the Balearic Islands Palma Mallorca Spain; 3 Emili Darder Health Centre Balearic Islands Health Service Palma Mallorca Spain; 4 Inca Hospital Balearic Islands Health Service Inca Mallorca Spain; 5 S’Escorxador Heath Centre Balearic Islands Health Service Palma Mallorca Spain; 6 Independent researcher Madrid Spain

**Keywords:** School Health Services, Primary Health Care, Adolescents, Secondary Education, Focus groups, Servicios de Salud Escolar, Atención Primaria, Adolescencia, Educación Secundaria, Grupos focales

## Abstract

**Background:**

*Consulta Joven* is a community intervention developed in primary care to address adolescents’ health needs. It offers open health consultations in secondary schools within a context of trust and confidentiality and is complemented by additional health promotion activities. This study explores the perceptions of professionals involved in the programme regarding challenges and key elements influencing its implementation.

**Methods:**

A qualitative, descriptive-interpretive approach study was conducted in primary care in Mallorca (Spain) in 2017. Participants were recruited by *Consulta Joven* coordinators using purposive theoretical sampling. Three focus groups were conducted with ten nurses, four physicians, and six teachers. Two researchers independently coded transcripts using a general inductive approach. Categories were developed through content analysis and refined through team discussion.

**Results:**

Four categories emerged: Perceived value and usefulness of *Consulta Joven*, Barriers to implementation, Key elements for programme development, and Proposals for improvement. Participants described *Consulta Joven* as an effective strategy to reach adolescents, promote healthy behaviours, and identify health problems before they seek medical care. Trust and confidentiality are highlighted as essential for its success, along with intersectoral collaboration with community stakeholders.

**Conclusions:**

Professionals perceive *Consulta Joven* as a useful intervention to adolescent health promotion. Strengthening institutional support and collaboration between the health and education sectors, and introducing the programme at earlier educational stages, may enhance its preventive impact.

## RESUMEN

**Fundamento:**

Atención Primaria desarrolló *Consulta Joven* como una intervención comunitaria en adolescentes, ofreciendo consultas de salud abiertas en centros de educación secundaria en un ambiente de confianza y confidencialidad; se complementa con otras actividades de promoción de la salud. El objetivo es explorar las percepciones de los profesionales involucrados en *Consulta Joven* sobre las dificultades y aspectos clave para su funcionamiento.

**Métodos:**

Estudio cualitativo, descriptivo-interpretativo, realizado en Atención Primaria (Mallorca, España) en 2017. Los participantes fueron reclutados por los responsables de la *Consulta Joven* tras un muestreo intencional teórico. Se realizaron tres grupos focales con personal de enfermería (n=10), medicina (n=4) y docente (n=6). Dos investigadores codificaron independientemente las transcripciones utilizando un enfoque inductivo general. Se realizó análisis del contenido y se discutieron los resultados hasta acordar las categorías incluidas.

**Resultados:**

Se detectaron cuatro categorías: Evaluación y utilidad, Dificultades de implementación, Aspectos clave para su desarrollo, y Propuestas de mejora para su desarrollo. Para los profesionales, *Consulta Joven* fue una forma eficaz de llegar a los adolescentes, promover su salud y detectar problemas antes de requerir atención sanitaria. Las claves de su éxito son confianza y confidencialidad, así como la colaboración intersectorial con los actores comunitarios.

**Conclusiones:**

Los profesionales perciben *Consulta Joven* como una intervención eficaz para promocionar la salud de los adolescentes. Señalaron la necesidad de iniciar Consulta Joven en educación primaria, contando con el apoyo institucional necesario para realizarla en condiciones óptimas y maximizar su efecto preventivo.

## INTRODUCTION

Adolescence is a transitional stage between childhood and adulthood during which individuals shape their identity and lifestyle. Although most Spanish adolescents are generally healthy^1^, they may adopt unhealthy habits and engage in health-risk behaviours. Consequently, international^2^, national^3^^,^^4^, and local^5^^,^^6^ organisations advocate for health promotion and education (HPE) strategies targeting adolescents in education centres (ECs)^2^. The school setting is widely recognised as an optimal environment for implementing health interventions^7^. Accordingly, many European countries have developed school-based HPE policies and programmes. The Schools for Health in Europe network exemplifies this approach, supporting the implementation of HPE initiatives in educational settings worldwide^7^. In Spain, compulsory education until the age of 16 facilitates equitable access to such interventions. In addition, a well-established primary healthcare network contributes to their delivery^4^. Several programmes have been developed, including “*Forma Joven*” in Andalusia, “*Salut i Escola*” in Catalonia, and the Aragonese Network of Health-Promoting Schools^8^^-^^10^. Previous evaluations suggest these initiatives are effective in reaching adolescents, enabling early detection of health problems, and supporting community health strategies.

In this context, *Consulta Joven* (CJ) was launched in Mallorca (Spain) in the 2004-2005 academic year to strengthen collaboration between primary care and the educational community (students, teachers, and families), as well as other stakeholders^6^. The programme is based on a holistic approach to health and promotes healthy lifestyle among adolescents. CJ provides open-access counselling sessions in secondary schools, typically on a weekly basis, delivered by healthcare professionals (HCPs) - usually a nurse - from the reference health centre (HC). These sessions address students’ health concerns in a confidential setting and may be conducted individually or in groups. They are complemented by additional HPE activities, such as workshops and educational sessions. Since the 2014-2015 academic year, ECs may also participate in the Health Promoting Schools Programme, which supports a systematic approach to health and well-being^11^.

The implementation of CJ has progressively expanded, from three participating HCs/ECs in 2004-2005 to 39 HCs (85%) and 65 ECs (44%) in 2023-2024^12^.

Annual evaluations include descriptive data on consultations, reasons for attendance, referrals, and HPE activities^12^. However, to our knowledge, no qualitative evaluation has explored the perspectives of key stakeholders (adolescents, families, and professionals).

This study aims to explore the perceptions of healthcare and education professionals regarding CJ, including perceived barriers, key factors influencing its implementation, and proposed areas for improvement.

## METHODS

This qualitative study, adopting an interpretive approach, was conducted in primary care settings in Mallorca, Spain. Data were collected at the end of the 2016-2017 academic year.

The study forms part of a participatory evaluation^13^ of CJ, aimed at reflecting on and improving the intervention from the perspective of its stakeholders. The present work focused on the education and HCPs responsible for its implementation, with the additional objective of enhancing their engagement and sense of ownership. A qualitative methodology was employed to explore participants’ perceptions, experiences, and expectations.

Participants were selected using purposive sampling to ensure variation in professional roles (physicians, nurses, and educators) and settings (rural and urban), complemented by snowball recruitment.Three focus groups were conducted with a total of 20 professionals (90% female): ten nurses, four general practitioners, and six teachers and/or school counsellors. Nurses constituted the largest groups, reflecting their central role in delivering CJ.

Focus groups were conducted during the 2016-2017 academic year and lasted approximately between 40 and 90 minutes (90 for nurses, 70 for teachers and 40 for general practitioners). Each session was moderated by a member of the research team and accompanied by an observer. Moderators were family medicine residents trained in qualitative research and not involved in CJ. The observer was a research technician with expertise in qualitative methodology. All focus groups followed a common semi-structured guide (Table 1) developed based on the study objectives, applied flexibly, and refined during fieldwork. All sessions were recorded and transcribed with participants’ consent.

The Primary Care Research Commission of Mallorca approved the study. It was conducted in collaboration with primary care centres and secondary schools involved in CJ. Participation was voluntary, with no financial compensation. All participants were informed about the study objectives, provided written informed consent, and were assured of confidentiality through anonymized coding.

**Table 1:** Focus group guide

*General perceptions*
1. What are your views of *Consulta Joven*?
2. What is your overall impression of the programme?
*Perceived usefulness*
1. In your opinion, what is *Consulta Joven* useful for? Who benefits from it? Does it add value to the centre?
2. Does *Consulta Joven* facilitate the identification of health or social problems? How are these managed when detected?
3. Does it help adolescents resolve their concerns? Does it affect the routine functioning of the centre?
4. Does *Consulta Joven* contribute to health promotion and health education among adolescents?
*Motivation*
1. What factors motivate or demotivate you?
2. Which professionals are most involved within the primary care team? And within the educational setting? Why?
3. What challenges do you encounter (e.g., communication or organizational difficulties?)
*Support*
1. Do you receive support from other professionals? From management or institutional bodies?
2. Are the necessary resources and material readily available?
*Training*
1. Have you received any specific training related to *Consulta Joven*?
2. Do you consider the training necessary?
Future perspectives
How could *Consulta Joven* be improved?
Are there any additional comments or experiences you would like to share?

### Data analysis

Two researchers independently coded the transcripts using a general inductive approach, followed by thematic content analysis, Analytical triangulation was ensured by involving one researcher familiar with the programme and another external to it. Categories were iteratively refined through team discussions^2^, creating a coherent category framework^14^ (Table 2).

**Table 2:** Map of categories

*1. Evaluation and perceived usefulness of Consulta Joven*
a. Overall assessment
b. Reasons for consultation
c. Referrals to other services
d. Impact as a community-based approach
e. Health education in schools
f. Professional motivation regarding *Consulta Joven*
*2. Barriers to the implementation of Consulta Joven*
a. Barriers related to access and healthcare professionals
b. Barriers related to adolescents and their families
*3. Key factors for the implementation of Consulta Joven*
*4. Proposals for improvement*

To enhance the credibility, preliminary findings were shared with participants, who were invited (questionnaire) to assess the interpretation of results. This validation process included eleven health care professionals and nine teachers.

No gender-based analysis was conducted; therefore, potential differences were not explored. No gender-related differences emerged among male participants that warranted specific reporting.

## RESULTS

The findings are presented according to the final category framework shown in Table 2. Verbatim quotations from participants are provided in Appendices 1 - 3 (translated from Spanish).

### Evaluation and perceived usefulness of *Consulta Joven*

#### Overall assessment

Both teachers and HCPs reported a positive overall assessment of CJ. Teachers described it is beneficial for both staff and students, as it provides a safe space where adolescents can raise concerns that they may not feel comfortable discussing with teachers or family members. Furthermore, participants perceived that the programme targeted a population group that may be at increased health risks and has limited access to healthcare services.

*I think it is very positive that there is CJ at the centre, firstly because the children ask questions that they do not ask us or their parents... then at the level of training for both students and teachers, talks, workshops..*. (Teacher)

*There is demand, there is interest, there are consultations... because of their age we write them off as not being concerned and they are concerned about their health.* (Nurse)

Teachers highlighted improved access to health support for younger students, who may have limited ability to attend health centres independently. They also highlighted that external professionals enhance credibility and foster trust among adolescents.

*We highly value that health personnel come to the educational centre... it should be considered they are minors, and they cannot go to the health centre on their own. (*Teacher)

*The fact that external people, health professionals, come here gives credibility and confidentiality is very important.* (Teacher)

*They are experienced people, so they already know what the specific problems of the pupils are. I think it is an advantage that they have experience with pupils of these ages.* (Teacher)

Nurses emphasised that the CJ is primarily a health promotion and education intervention, distinct from routine primary care consultations, which are more focused on acute conditions. They underline its role in improving adolescents’ knowledge and supporting informed decision-making.

*In the educational centre, the consultation is for health promotion and education.* (Nurse)

*Our role is also to give tools to help them learn to make decisions. These decisions must be taken with a series of criteria and that they will have consequences that they must assume... I think we must work a lot on this aspect.* (Nurse)

HCPs also highlighted the creation of a trusted and confidential environment, enabling adolescents to express themselves freely and discuss sensitive topics. Some participants noted that positive initial experiences often lead adolescents to encourage their peers to attend.

*The fact that external people, health professionals, come here gives credibility and confidentiality is very important*. (Teacher)

*They place a lot of value on what happens there (the consultation) as it is a space where they (the adolescents) can express themselves freely. The trust and closeness that is generated is very important.* (Nurse)

*When the first consultation goes well for the student, they usually bring friends.* (Family physician)

#### Reasons for consultation

The most frequent reasons for consultation were related to sexual health. However, participants reported a growing number of sessions addressing psychosocial issues, including emotional distress, interpersonal relationships, bullying, and violence.

*Sexuality.... not to mention, it is the part of the consultations we have most; doubts at a sexual level, particularly of discovering about themselves; many questions are about what the female genitalia are like, how they work....* (Family physician)

*At the beginning - I have been involved with CJ for 4 years - issues on relationships with parents or between peers were practically none... however, over time more and more they come to talk about relationships with parents, siblings, friends ....* (Nurse)

HCP also described managing complex and sensitive cases, such as eating disorders, sexual abuse, bullying, often requiring culturally sensitive approaches.

*The cultural part is very important; we have Muslims there, people from different cultures... with different ways of seeing it...* (Family physician)

Several participants reported that CJ facilitates the early detection of serious health concerns, including mental health disorders, self-harm, and abuse, which might otherwise remain hidden or be identified later within the healthcare system. In such cases, coordination with families and other professionals is necessary, while carefully maintaining adolescents’ trust and confidentiality.

*There have been cases detected that are very serious, exaggeratedly serious. They (health professionals) help us, in serious mental health problems, they help us, there is a vacuum in this area.* (Teacher)

*You get very delicate things, abuse... you get to see many situations...* (Family physician)

*Important things are detected; for example, some adolescent, with homosexuality issue have attempts at self-harm. We refer them to the child and adolescent mental health unit...in other words, I believe that many things are detected.* (Family physician)

#### Referrals to other services

In some cases, identified problems require referral to other services. Most referrals were made to primary care professionals, with fewer directed to mental health services or social workers. Complex cases occasionally required legal consultations. Teachers noted that the involvement of HCPs facilitated case management and improved decision-making regarding appropriate referral pathways.

In conflicts between the adolescent and their families, referral processes became more complex but also created mediation opportunities.

*Although consultations are aimed at health promotion, we must know when to refer...and in principle it is to those responsible for health (family physician, paediatrician, nurse) we must also work with the counselling and social services team*. (Nurse)

*It improves the effectiveness of case resolution because it helps us to know where to go*. (Teacher)

#### Consulta Joven as a community approach

Nurses emphasised the intersectoral and community-based nature of CJ, highlighting collaboration with multiple stakeholders. They also underscored their role as health promoters within community care.

Teachers noted that the programme helped adolescents identify reliable sources of health information and had a broader dissemination effect, as knowledge acquired by students was often shared with peers and family members.

*We work with the police guardian, with street educators, with social services...* (Nurse)

*Each student has a family, it is a way for the health information to be amplified, it is a cascade, it reaches 4 or 5 people in their family, they just arrive and say one thing that they have learned...* (Teacher)

#### Health education in schools

In all the ECs, consultations were complemented by health education workshops for students and teachers, which were considered essential. Teacher involvement helped ensure ongoing engagement, as content introduced enabled reinforcement through classroom activities or tutorials.

*More and more teachers are involved, they give you dynamics to work with and if they attend (the workshops) they give continuity in their tutorial action plan.* (Nurse)

*The workshops, planned at the beginning of the school year, are very good, there are many (nutrition, affectivity, sexuality, piercings, bullying, smoking awareness tables, personal hygiene...).* (Teacher)

*In cases of students with diabetes, epilepsy, etc., we had coordinated training not only for the student, but also for the staff ... first aid...* (Teacher)

#### Motivation of professionals

Overall, participants were motivated, particularly nurses. However, some physicians expressed frustration when scheduled consultations were underutilised by adolescents.

Teachers indicated that programme maturity influenced motivation: while initial enthusiasm may decline over time, it can be renewed through ongoing adaptation and the integration of complementary activities such as workshops. Nurses emphasized that CJ encompasses a broader set of activities beyond individual consultations.

*Very beneficial for them (the adolescents) as well as for us.* (Nurse)

*...the organisation in the educational centre...when there are no consultations and going to the centre by health professionals is a waste of time.* (Family physician)

*It evolves over the years; very good at the beginning, and then there is a decline...the demands of workshops have increased. It must be nurtured in one way or another. Alternating workshops and consultation is doing well.* (Teacher)

### Barriers to the implementation of *Consulta Joven*

#### Barriers related to access and professionals

Participants identified inequitable access as a key limitation, as CJ is not implemented in all schools (Appendix I, a.i). HCPs also reported resistance among some staff members towards this type of programme and suggested that it should be formally integrated into the services’ portfolio rather than being voluntary (Appendix I, b.i-iii).

Teachers highlighted the time constraints during work hours as a barrier to participation in the activities of the programme (Appendix I, b.iv). Physicians noted that some teachers were reluctant to facilitate adolescents’ attendance at consultations or allocate time for health promotion activities (Appendix I, b.v). Conversely, teachers acknowledged the difficulties HCPs face in attending the EC regularly (Appendix I, b.vi).

#### Barriers related to adolescents and their families

Some physicians noted that adolescents’ accounts may sometimes be exaggerated or biased, requiring careful interpretation. Another key challenge identified by HCPs was limited engagements with families (Appendix I, c.i-iii), which can hinder comprehensive management of identified issues.

### Key factors for effective implementation of *Consulta Joven*

Participants emphasised the importance of involvement from management teams in both ECs and primary health settings (Appendix II, a.i-ii). Integration into the school’s educational framework was considered essential. Accreditation as a health-promoting school was also perceived as facilitating implementation (Appendix II, b.i-ii).

The existence of an intersectoral health committee within schools was highlighted as a key facilitating factor, supporting coordination and adaptation to local needs. Participants valued the inclusion of family and student representatives, although their participation was reported to be limited. School counsellors were identified as key actors due to their close knowledge of students and their role in facilitating coordination and case management (Appendix II, b.i-ii).

### Proposals for improvement

Participants proposed strategies to strengthen CJ. These included increasing institutional support through adequate allocation of staff and time, extending the programme to all schools, and introducing it earlier, particularly in the final years of primary education (ages 11-12).

Additional recommendations included strengthening intersectoral coordination, improving integration with school activities, ensuring appropriate consultation spaces, and increasing the frequency of consultations. Participants also highlighted the need to update educational materials, enhance programme visibility, provide ongoing training for professionals, and implement systematic evaluation strategies to demonstrate the program’s impact (Appendix III).

## DISCUSSION

Professionals report a positive appraisal of the program, which improves access to health services to adolescents and facilitates both health education and the early detection of problems that might otherwise reach the healthcare system at a later stage**.**

Several studies have demonstrated the effectiveness of school-based programmes targeting different health problems (e.g., anxiety, obesity)^15^ as well as those adopting a whole-school approach^16^. Previous research, such as that by Leite *et al*., together with PAHO/WHO guidelines^3^ and the European SHE programme^7^, highlights the successful implementation of school-based initiatives that are not limited to a welfare approach but are coordinated with referral health services, and adopt a salutogenic perspective^17^^,^^18^.

The value of CJ lies in the involvement of primary care professionals as referral providers who conduct consultations within the EC, thereby promoting comprehensive and community-based care. Moreover, locating consultations within the HC facilitates adolescents’ access to health services without the direct involvement of their parents^19^^,^^20^. This is particularly important given the challenge of addressing adolescent health issues in primary care settings, especially among minors^21^, and helps to overcome barriers to accessing health services^17^^,^^19^^-^^22^. Adolescents often have limited access to guidance on healthy behaviours^1^^,^^23^, partly due to the predominantly clinical and pathogenic orientation of these services^16^^,^^23^^,^^24^.

In addition, *CJ* provides a confidential space within EC where adolescents can seek advice, in line with WHO recommendations^25^^,^^26^. Professionals also emphasized the importance of close coordination and follow-up between health professionals delivering complementary activities (e.g., workshops, sessions) and teaching staff. This collaboration contributes to improving adolescents’ knowledge and skills for making healthier decisions^24^^-^^26^.

Although the primary aim of the consultation is health promotion, it also enables the identification of problems requiring intervention and referral to other services. Thus, the consultation becomes a trusted setting for the early detection of health issues that might otherwise reach the healthcare system at a later stage^17^^,^^22^^,^^27^^,^^28^. The trust adolescents place in professionals facilitates timely intervention and improves communication with families.

Intersectoral collaboration^1^ is essential for the success of CJ. As noted by Leite *et al*.^29^, the active involvement of both families and adolescents is critical, as awareness of the programme is necessary for meaningful participation. Collaboration with counsellors is essential for the proper development of the consultation given their role in student care and support^30^.

Since the COVID-19 pandemic, health and education authorities in the Balearic Islands have established that all ECs should have a health commission to facilitate coordination between sectors^31^. This recommendation had already been proposed at the early stages of CJ implementation in Mallorca^6^, although initially its adoption depended on the voluntary engagements of individual ECs. Such coordination mechanisms are considered essential for strengthening joint efforts and ensuring comprehensive adolescent care^2^.

Despite its clear benefits, professionals identified several challenges, many of which are common to school health programmes. One major issue is inequity in implementation, as not all ECs are able to adopt the programmes; its availability often depends on school leadership and the resources of primary care teams, despite being included in service portfolio and management contracts^32^.

Leadership of the consultation is primarily assumed by nurses^12^, as is common in many European school health programmes^3^. Although some physicians do not consider this activity part of their responsibilities, there has been increasing involvement of medical and paediatric professionals in recent years, possibly reflecting enhanced training in community health within the family and community medicine specialty in Spain^33^. Another reported challenge is the limited transmission of health messages to families. As noted in previous studies, parental engagement tends to decline in secondary education, and adolescents themselves present communication challenges within families. Nevertheless, participants believe that the content addressed in CJ influences both families and peers. In this regard, identifying adolescents with strong communication skills and engaging them as peer educators may help promote behavioural change^17^.

Key factors for the successful implementation of CJ include the involvement of SC and EC management teams, integration within the school project, intersectoral coordination, and the participation of families and students. These findings are consistent with previous literature, including reviews by Webster *et al*.^34^^)^ and Bennasar Veny *et al*.^35^, which emphasize the crucial role of school leadership in the success of school health programmes^29^^,^^36^. High staff turnover in both in education and, particularly, primary care further underscores the importance of management teams in ensuring continuity, facilitating team reorganisation and supporting the training of new professionals^36^. Additionally, excessive clinical workload limits the time professionals can dedicate to community-based activities^2^.

As in other studies, participants emphasised the need for protected time within working hours to carry out CJ activities. Training of HCPs, highlighted in WHO and SHE standards, is also viewed as a key strength^7^^,^^35^.

One of the main strengths of this study is the inclusion of the perspective of the professionals involved, who showed considerable agreement in their assessments of strengths, challenges, and areas for improvement^12^. Furthermore, no similar qualitative evaluations of comparable programmes in other autonomous communities were identified.

Several limitations should be noted. All participants were linked to CJ, as they belonged to centres where the programme is implemented; therefore, the perspectives of non-participating professionals were not captured. Furthermore, data collection took place shortly before the COVID-19 pandemic. Although the study has not been updated, subsequent meetings with the professionals have underscored the need to further strengthen CJ in response to the increase in adolescent mental health problems following the pandemic. Professionals report identifying more severe cases, reinforcing the importance of a comprehensive and interdisciplinary approach.

This study is complemented by ongoing research conducted by the authors exploring the perspectives of students and their families, which is currently under review.

In conclusion, professionals consider that CJ facilitates engagement with adolescents, supports informed decision-making regarding their health, and enables the early detection of problems. Both health and education professionals view the programme as useful and necessary and emphasise the importance of intersectoral collaboration with community stakeholders. However, they also highlight the need to extend CJ to primary education and to ensure adequate institutional support for its implementation under optimal conditions.

## Data Availability

The data that support the findings of this study are available from the corresponding author, [MCVT], upon reasonable request.

## APPENDIX I

**Table:** Difficulties in implementing the *Consulta Joven*. Textual quotations for the different subcategories

a) Institution-related
i*. There are new institutes that are asking us, in the area, we do not cover all of them. They complain because we do not go... they like it (family physician).*
b) Related to professionals
i*. We started with many difficulties (with colleagues at the health centre), but it gradually improved (family physician).*
ii*. A very involved family physician had to leave, due to peer pressure and pressure from the practice, very sad, she was very involved and highly valued... (nurse).*
iii*. We must put an end to the distinction between those who want to participate and those who do not...I am a nurse at the health centre, and this is one of my function (nurse).*
iv*. If you get involved (in youth work), you need to dedicate extra hours. Sometimes there are problems because we cannot do all everything; we must adapt and organise... (teacher).*
v*. They (teachers) have motivation, but lack organisation. It is not part of their project; they consider it something additional (family physician).*
vi*. We ask for greater willingness within working hours, and more resources - availability of time - to be able to provide better support (teacher).*
c) Related to adolescents and their families
i*. They tell you many things that you do not know whether they are true or not, issues of maltreatment, abuse... there are serious situations.,, and you see them... but there are also adolescents who lie frequently. When you have to report to child protection services... all these issues... you reflect on them extensively... (family physician).*
ii*. We wanted to set up monitoring through appointments, but then attendance decreased... I proposed that whoever wants to come could do so, and if a problem arises, we can address it...because if we put obstacles to participation, it works against it (teacher).*
iii*. Reaching parents is more difficult... (nurse).*

## APPENDIX II

**Table:** Key aspects for the development of *consulta joven*. Textual quotations for the different subcategories

a) Involvement of school and health centre management teams
i*. We (the management team) must make the most of this resource. We have to be attentive, talk to the tutors, and plan the workshops... Inform parents via the website, inform new pupils... (teacher). It should reach all ECs.*
ii*. The nursing coordinator (or person in charge) is very important; they play a key role as catalysts in ensuring implementation (nurse).*
b) *Consula Joven* is integrated in the school’s educational project
i*. If it is integrated (into the educational project) it works... We used to do it... now it has been structured, with the health promotion centres. We have improved, I believe so... (teacher).*
ii*. It is a good working tool, all actions are integrated... also, since we became a health promotion centre, it works better. They come every week, and we perceive improvements (teacher).*
c) Existence of an intersectoral health commission within the educational centre
i*. At the beginning of the school year, we do the planning together, it is very positive. They implement many activities (nutrition, affectivity, sexuality, healthy breakfasts, piercings, bulling, tobacco awareness activities...) ... these are initiatives that work... (teacher).*
ii*. We work closely with the counsellor; they are usually aware of the cases we report to them. They are informed, except for a few specific cases (nurse).*

## APPENDIX III

**Table:** Improvement proposals for the development of *Consulta Joven*

*Consulta Joven* in all educational establishments
*It should be implemented in a generalised way through an agreement with the Ministry of Education, to be carried out in all secondary schools with a well-planned protocol (nurse). The health sector must understand that this benefits of the population (teacher).*
Extend to 5th-6th grades of primary education
*It should start earlier, in primary school (family physician).*
Improve nurse-to-school ratios according to the educational centres in the basic health area
*We need nurse-to-school-ratios based on the number of schools in the area; otherwise, we cannot cope (nurse). Sometimes there are problems because they (nurses) cannot do everything, we must adapt, especially when there are budget cuts in healthcare... (teacher).*
Provide time during working hours to prepare sessions, workshops and related activities
*We need to increase staffing in line with the health education and community activities we carry out (nurse). ...it involves a great deal of work; workshops and meetings must be prepared... almost always outside working hours... (nurse). We cannot do more than we already do; we need more time and must look for gaps. Sometimes organisation suffers because everything must be done in a hurry (teacher).*
Ensure activities are adapted to the needs of the centre and aimed at developing skills, not only knowledge, incorporating a health assets perspective
*It is important to adapt to the needs of the centre... they asked us to carry out an intervention on bullying instead of working of nutrition due to situations of significant conflict, which caused distress among teachers; we delivered 12 interactive workshops... (nurse). Efforts should focus on ensuring that adolescents acquire skills, not just knowledge (nurse).*
Encourage intersectoral work through the health commission at the educational centre. This facilitates planning according to the real needs of the educational community, case resolution, and the coordination of activities
*The commission decides which workshops, topics, and facilitators will be involved at the beginning or end of the school year for the following course (nurse). The health commission is useful for gathering information from all stakeholders (teachers).*
Appointment scheduling helps health professionals organise their time but may hinder adolescents’ access
*Appointments allow better management of schedules (family physician, nurse) and influence whether professionals attend schools depending on availability (family physician).*
*Walk-in consultations gives students more freedom to ask questions (nurse).*
Encourage tutoring hours to deepen and ensure continuity of topics covered in workshops or consultations.
*It is very important that workshops take place during tutoring hours, as these allow for follow-up and continuity (teacher). Whenever possible, workshops should be organised jointly with teachers, so that they can continue the topics in tutorials and classes... (nurse).*
Improve dissemination, both within (class by class, posters...) and outside (social networks, websites, school and health centre teams...) the school
*Dissemination needs improvement: professionals go from class to class, introduce themselves, and explain what students can consult them about... (teacher).*
Update educational materials with an accessible repository
*Yes, we have material, although we would like to have more visual and updated resources (nurse).*
Improve professional training, mainly on legal aspects, bullying, gender-based violence, mental health, bereavement, etc.
*One form of training is observational learning - seeing how another nurse works (nurse). Training has been provided on different topics almost every year, but it is necessary to continue and expand; ongoing professional development is key (nurse).*
Evaluate to provide evidence of effectiveness
*We must conduct research so that those who need evidence can believe in it. We are already convinced because we see it impact, but some professionals require formal evidence (nurse).*

## References

[1] Ministerio de Sanidad, Instituto Nacional de Estadística Spanish National Health Survey 2017.

[2] Pan American Health Organization (2022). Making all schools health promoting: global guidelines and indicators.

[3] Ministerio de Educación, Formación Profesional y Deportes School Health - SGCTIE.

[4] Blanco López JL, Miguel Pérez V, Crespo Balcones JL, Echeita Blanco M, Mendieta Garrote E, Martínez Duarte M (2017). Strategic plan for school health and healthy lifestyles (2016-2020).

[5] (2002). Agreement of the Government Council on the development of health-education programmes in schools. Butlletí Oficial de les Illes Balears.

[6] Ordinas Vaquer M, Vidal Thomàs C, Mayol Quetglas C, Mora Cerdá J, Sánchez Sansó F, Font Oliver MA Health education in schools through intersectoral projects 2010.

[7] Paakkari L, Simovska V, Pedersen U, Schulz A, Lopez L, Von Seelen J Teachers' materials: Learning about health and health promotion in schools. Key concepts and activities. Schools for Health in Europe. October 2019.

[8] Consejería de Salud y Consumo, Junta de Andalucía Forma Joven-Professional Network.

[9] Generalitat de Catalunya, Agència de Salut Pública de Catalunya Health and school.

[10] Gobierno de Aragón Red Aragonesa de Escuelas Promotoras de Salud (RAEPS) [Aragonese Network of Health-Promoting Schools].

[11] Govern de les Illes Balears Programa de Centres Educatius Promotors de la Salut (CEPS) [Health-Promoting Educational Centres Programme].

[12] Gerència d'Atenció Primària de Mallorca (Servei de Salut de les Illes Balears) Evaluation of "Consulta Jove" #APMallorca.

[13] Guijt I (2014). Participatory approaches. Methodological briefs: impact evaluation 5.

[14] Vidal-Thomàs C, March Llull S, Cacereño Jiménez MB, Sempere Andrés P, Urbina Sampedro E (2017). Participatory evaluation of the "Consulta Jove" Programme: professionals' assessment.

[15] Levinson J, Kohl K, Baltag V, Ross DA (2019). Investigating the effectiveness of school health services delivered by a health provider: a systematic review of systematic reviews. PLoS One.

[16] Stewart-Brown S (2006). What is the evidence on school health promotion in improving health or preventing disease and, specifically, what is the effectiveness of the health promoting schools approach? Health Evidence Network report.

[17] Anderson JE, Corrine M, Lowen CA (2010). Connecting youth with health services: systematic review. Can Fam Physician.

[18] Richler A, Sjunnestrand M, Strandh M, Hasson H (2022). Implementing school-based mental health services: A scoping review of the literature summarizing the factors that affect implementation. Int J Environ Res Public Health.

[19] Jacobson LD, Mellanby AR, Donovan C, Taylor B, Tripp JH, Adolescent Working Group R (2000). Teenagers' views on general practice consultation and other medical advice. Fam Pract.

[20] Quiroz-Cardenas F, López-Gil JF (2025). The role of school-based health centers in adolescent well-being: a call for action. Front Public Health.

[21] Ramis Roca MR, Cáceres Teijeiro Y, Vidal Thomàs MC, Núñez Jiménez C, Carandell Jägger E, Lafau Marchena O (2022). Legal aspects of healthcare for minors within the "Consulta Jove" framework.

[22] Bains RM, Diallo AF (2016). Mental health services in school-based health centers: systematic review. J Sch Nurs.

[23] Goicolea I, Aguiló E, Madrid J (2015). Is youth-friendly primary care feasible in Spain?. Gac Sanit.

[24] World Health Organization (2015). Improving the quality of adolescent health care using a standards-based approach.

[25] World Health Organization (2015). Global standards for quality health-care services for adolescents: A guide to implement a standards-driven approach to improve the quality of health-care service for adolescents.

[26] The Society for Adolescent Health and Medicine (2025). Confidential healthcare for adolescent minors and young adults: A position paper of the society for adolescent health and medicine. J Adolesc Health.

[27] Farias ICV de, Franco de Sá RMP, Figueiredo N, Menezes A (2016). Analysis of intersectorality in the School Health Program. Rev Bras Educ Med.

[28] Bersamin MM, Fisher DA, Gaidus AJ, Gruenewald PJ (2016). School-based health centers' presence: The role of school and community factors. Am J Prev Med.

[29] Leite CT, Machado M de F, Vieira RP, Marinho MN, Monteiro CF (2015). The School Health Program: teachers' perceptions. Invest Educ Enferm.

[30] Cobos Cedillo A (2016). Educational guidance is essential to improve the quality of the education system. Educar y orientar.

[31] Govern de les Illes Balears (2021). Resolución conjunta de la Conselleria d'Educació i Formació Professional y la Conselleria de Salut i Consum. [Joint Resolution exceptional prevention and organizational measures for non-university educational centres, 2021-2022]. Butlletí Oficial de les Illes Balears.

[32] Gerència d'Atenció Primària de Mallorca Service portfolio.

[33] Unitat Docent Multiprofessional d'Atenció Familiar i Comunitària (Mallorca) Teaching Unit webpage.

[34] Webster CA, Glascoe G, Moore C, Dauenhauer B, Egan CA, Russ LB (2020). Recommendations for administrators' involvement in school-based health promotion: a scoping review. Int J Environ Res Public Health.

[35] Bennasar Veny M, González Torrente S, Pericás Beltrán J, Segui González P (2009). Evaluation of the implementation of a Teen Clinic in a secondary school. Enferm Comun.

[36] Seguí González P, Gonzalez Torrente S, Bennassar Veny M, Pericás Beltrán J (2010). Analysis of a health-promotion activity using the EFQM quality model. Rev Paraninfo Digit.

